# Dicer prevents genome instability in response to replication stress

**DOI:** 10.18632/oncotarget.27034

**Published:** 2019-07-09

**Authors:** Michalis Fragkos, Viviana Barra, Tom Egger, Benoit Bordignon, Delphine Lemacon, Valeria Naim, Arnaud Coquelle

**Affiliations:** ^1^ IRCM, Institut de Recherche en Cancérologie de Montpellier, INSERM U1194, Université de Montpellier, Institut Régional du Cancer de Montpellier, Montpellier, France; ^2^ Laboratory of Genetic Instability and Oncogenesis, UMR 8200 CNRS, University Paris-Sud, Gustave Roussy, Villejuif, France; ^3^ Present address: Department of Biochemistry and Molecular Biology, Doisy Research Center, St. Louis, MO, USA; ^*^ These authors contributed equally to this work; ^#^ Lead contact

**Keywords:** replication stress, common fragile sites, Dicer, genomic instability

## Abstract

Dicer, an endoribonuclease best-known for its role in microRNA biogenesis and RNA interference pathway, has been shown to play a role in the DNA damage response and repair of double-stranded DNA breaks (DSBs) in mammalian cells. However, it remains unknown whether Dicer is also important to preserve genome integrity upon replication stress. To address this question, we focused our study on common fragile sites (CFSs), which are susceptible to breakage after replication stress. We show that inhibition of the Dicer pathway leads to an increase in CFS expression upon induction of replication stress and to an accumulation of 53BP1 nuclear bodies, indicating transmission of replication-associated damage. We also show that in absence of a functional Dicer or Drosha, the assembly into nuclear foci of the Fanconi anemia (FA) protein FANCD2 and of the replication and checkpoint factor TopBP1 in response to replication stress is impaired, and the activation of the S-phase checkpoint is defective. Based on these results, we propose that Dicer pre-vents genomic instability after replication stress, by allowing the proper recruitment to stalled forks of proteins that are necessary to maintain replication fork stability and activate the S-phase checkpoint, thus limiting cells from proceeding into mitosis with under-replicated DNA.

## INTRODUCTION

DNA replication is a fundamental cellular process that needs to be tightly regulated to maintain the genetic information through generations. Any challenge to this process slowing down or stalling the replication fork constitutes a Replication Stress (RS) that can have severe implications for genome stability, cell survival and even human diseases. Indeed, RS is considered a major driver of tumor initiation and progression. Some genomic regions, like Common fragile sites (CFSs), are particularly sensitive to RS and tend to form gaps or breaks on mitotic chromosomes after replication stress. CFSs are present in all individuals, lie in late-replicating regions and represent a significant source of genetic instability in cancer cells [1, 2]. The late replication and origin paucity of CFSs increase the risk of remaining incompletely replicated at the time of mitosis upon replication stress, when cells are unable of rescuing replication fork stalling/slowing and transcription-replication conflicts [1, 3–6]. When replication is challenged, the S-phase checkpoint is activated to protect stalled replication forks from collapsing and to delay cell cycle progression, preventing cells from entry into mitosis in presence of under-replicated or damaged DNA [7–10]. The activation of this checkpoint requires the relocalization into nuclear foci and the stepwise activation of proteins of the ATR-Chk1 and the Fanconi Anemia (FA) pathways [11], including Topoisomerase II Binding Protein 1 (TopBP1) and FANCD2 [12–15]. TopBP1 recruitment to sites of stalled replication forks or DNA damage is a crucial step for activating the ATR kinase and for its subsequent Chk1 phosphorylation [16]. FANCD2 is the key activated target of the FA pathway, deficient in Fanconi Anemia chromosomal instability and cancer-prone disorder, and is involved in replication stress response and inter-strand crosslink (ICL) repair [17]. Following replication stress or DNA damage, FANCD2 is mono-ubiquitylated by the FA core complex which promotes replication fork recovery and ICL repair, together with downstream components of the FA pathway, such as structure-specific endonucleases, translesion synthesis polymerases and homologous recombination proteins.

Non-coding RNAs (ncRNAs) have diverse roles in regulating transcriptional and post-transcriptional processes, chromatin dynamics, and cellular homeostasis [18, 19]. Micro RNAs (miRNAs) constitute a large class of small ncRNAs with a prominent role in regulation of gene expression [20]. They have also an essential role in cell viability and proliferation, but details of their mechanism of action remain largely unknown [21, 22]. The biogenesis of miRNAs is mediated by two main RNases: Drosha, which processes long primary precursor transcripts (pri-miRNAs) in the nucleus, and Dicer, which cleaves the secondary miRNA precursors (pre-miRNAs) in the cytoplasm to produce mature 21–25 nucleotide miRNAs [23, 24]. Intriguingly, it has been reported that some fragile sites and common breaking regions of both the human and murine genomes are located close to miRNA genes [25, 26]. In addition, cancer often correlates with alterations at ultra-conserved genomic areas expressing ncRNAs [27], suggesting that these RNAs might be involved in maintaining the stability of these sites under stress conditions. Other recent studies have shown that Dicer-processed RNA products are involved in DNA double-strand break (DSB) repair. These DDRNAs (DNA Damage response RNAs) are produced in the vicinity of the DSB sites and are used to facilitate the repair by mediating the binding of chromatin modifiers at the sites of damage [28–32]. RNA processing by both Dicer and Drosha following break induction in human cells promotes repair by both homologous recombination and non-homologous end joining [29, 32, 33]. In addition, Dicer itself, independently of its ribonuclease activity, has been recently discovered to be involved in Nucleotide Excision Repair (NER) by mediating the chromatin decompaction necessary to remove DNA lesions induced by UV light exposure [34–36].

Albeit the involvement of Dicer in DNA repair is quite clear and some progresses have been done to disclose the underlying mechanism, these aspects still remain elusive. Conventionally considered as a cytoplasmic protein, Dicer can relocalize to the nucleus thanks to its non-canonical Nuclear Localisation Signal (NLS) [37]. In fact, it has been demonstrated that in both humans and mice after DNA damage a fraction of Dicer is phosphorylated and accumulated in the nucleus to promote the recruitment of DNA repair factors [38, 39]. Strikingly, it has been shown that in yeast, Dicer promotes the release of RNA polymerase II from the termination site of highly transcribed genes and process the RNA:DNA hybrids resulting from transcription-replication collisions to maintain genome stability [40]. More recently, it has been demonstrated that Drosha-driven RNA:DNA hybrids localize around DSB sites, at the very beginning after H2A.X phosphorylation and ATM activation but before the accumulation of 53BP1 and DDRNAs - of the DNA damage response, suggesting that these structures have a key role in the DNA damage repair [41].

However, whether the Dicer pathway plays a role in the response to replication stress in higher eukaryotes is still not known. To investigate this, we used HCT116 cells, either Dicer-deficient or knocked down for Dicer and Drosha, to study the effect of the Dicer pathway impairment on CFS stability after induction of replication stress. Our data show that Dicer is required for maintaining genomic stability after replication stress and acts by promoting the recruitment of proteins involved in replication fork stability and S-phase checkpoint activation. Therefore, Dicer prevents cells from entering a mitosis with under-replicated DNA that would otherwise result in chromosome breaks and DNA damage transmission to daughter cells.

## RESULTS

### Inhibition of Dicer enhances genomic instability and fragility at CFSs

**Figure 1:**
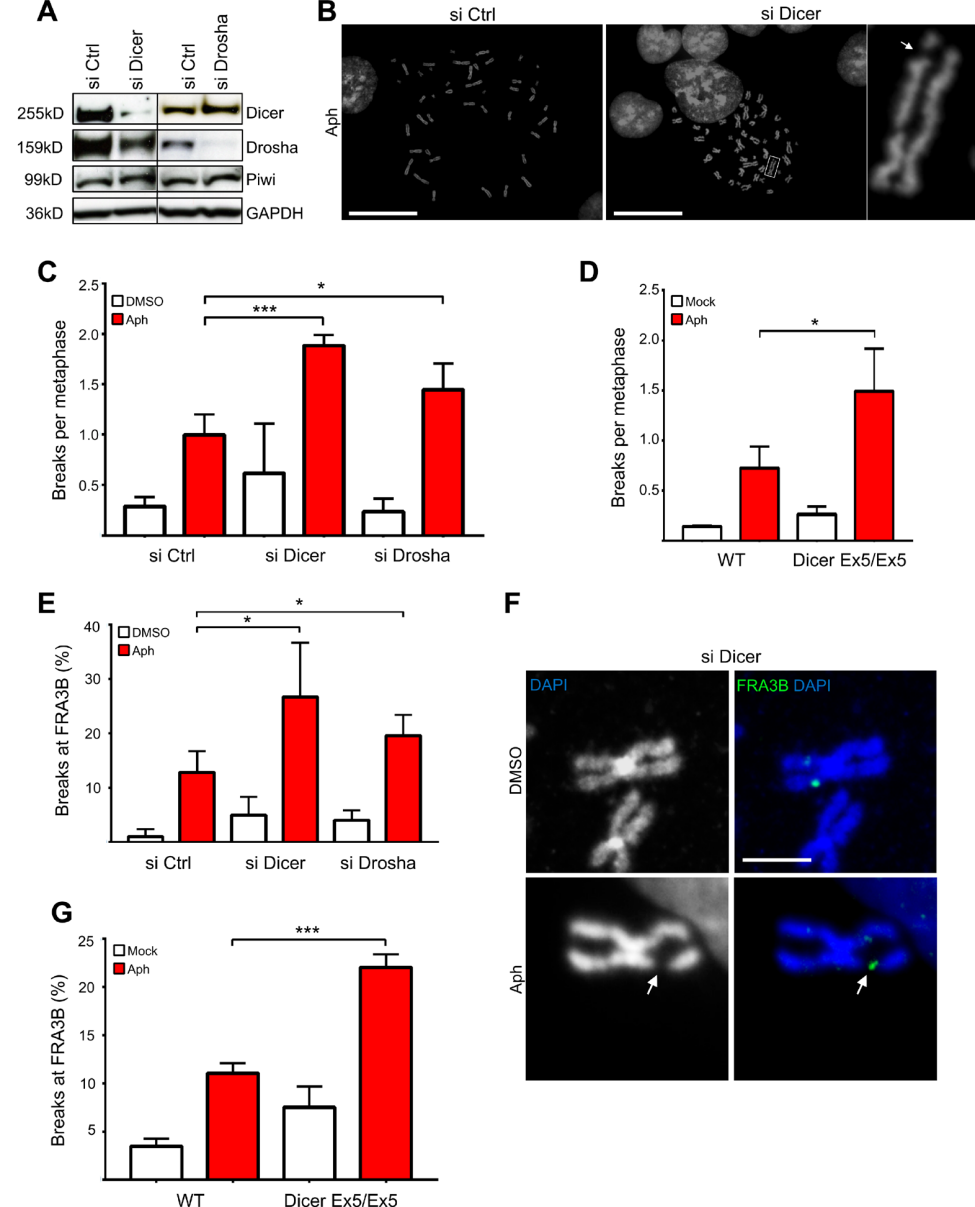
Inhibition of the Dicer pathway enhances genomic instability after replication stress. (**A**) Western blot showing Dicer and Drosha down-regulation after siRNA treatment in HCT116 cells. Piwi was used as an RNAi off-target control. GAPDH was used as a loading control. (**B**) Representative DAPI-stained metaphase spreads of cells treated with aphidicolin. (**C**) Inhibition of Dicer and Drosha enhances chromosome breakage after aphidicolin treatment. Dicer or Drosha expression were transiently inhibited by siRNA and cells were treated with aphidicolin to induce replication stress. Metaphase spreads were then prepared and analyzed by fluorescence microscopy. The graph presents the average number of chromosome breaks per metaphase. Error bars represent the SD of three independent experiments. Unpaired *t*-test: ^*^ *p *< 0.05, ^***^ *p *< 0.001. (**D**) Dicer-deficient HCT116 cells (Dicer Ex5/Ex5) show enhanced chromosomal fragility after aphidicolin treatment. WT and Dicer-deficient cells were treated with aphidicolin to induce chromosome breaks and were then processed for preparation of metaphase spreads. Metaphase spreads were stained with DAPI and analyzed by fluorescence microscopy. The graph presents the average number of chromosome breaks per metaphase. Error bars represent the SD of three independent experiments. Unpaired *t*-test: ^*^ *p *< 0.05. (**E**) Quantification of the FISH analysis for FRA3B, after inhibition of Dicer and Drosha by siRNA and induction of replication stress by aphidicolin. The graph presents the percentage of broken FRA3B sites. Error bars represent the SD of three independent experiments. Unpaired *t*-test: ^*^ *p *< 0.05. (**F**) Representative FISH images using a probe for the FRA3B fragile site. The white arrow indicates a characteristic chromosomal break. DNA was stained with DAPI and metaphase spreads were analyzed by fluorescence microscopy. (**G**) Graph presenting the results of the FISH analysis in Dicer-deficient and WT HCT116 cells after treatment with DMSO (−) or aphidicolin (+). The percentage of fragile FRA3B sites is shown. Error bars represent the SD of three independent experiments. Unpaired *t*-test: ^***^ *p*< 0.001.

To ascertain possible roles of the Dicer pathway in the DNA damage response induced by replication stalling, we investigated its function in genomic stability after treatment with mild doses of aphidicolin, an inhibitor of DNA polymerases, commonly used to induce breaks preferentially at CFSs [42]. To this end, we knocked down Dicer or Drosha in HCT116 cells, used as a model to study CFSs [43]. Cells were transfected with siRNA (small interfering RNA) pools targeting either Dicer or Drosha, and LacZ as a control. Dicer and Drosha protein levels were assayed by western blotting (Figure 1A). siRNAs targeting Dicer (siDicer) led to a significant decrease of Dicer expression and a partial decrease of Drosha expression; instead, siRNAs targeting Drosha (siDrosha) specifically reduced just the protein levels of Drosha. Both siRNA pools did not affect levels of Piwi, a protein that binds to another class of small non-coding RNAs called piRNAs, indicating the specificity of the RNAi [44]. Then, Dicer and Drosha-depleted cells were treated with aphidicolin to induce replication stress and metaphase spreads were analyzed. Inhibition of both Dicer and Drosha resulted in a significant increase in the average number of chromosomal breaks after aphidicolin treatment compared to the control (Figure 1B–1C).

To study further the role of Dicer in maintaining genome stability upon replication stress, we used an HCT116 cell line that is mutated in the RNA helicase domain of Dicer. These cells, hereafter named Dicer Ex5/Ex5, have an in-frame 129-bp homozygous insertion in Exon 5 of the Dicer gene, which inactivates its RNA helicase domain [45, 46]. This mutation was verified by PCR (Supplementary Figure 1A) and was shown to lead to a significant reduction in the levels of Dicer (Supplementary Figure 1B). These cells and their isogenic WT (Wild Type) Dicer counterparts were treated with aphidicolin and metaphase spreads were obtained as described above. Consistent with the results following Dicer knock down, the frequency of chromosome breaks after replication stress was enhanced in Dicer Ex5/Ex5 cells (Figure 1D), further supporting a role for Dicer in maintaining genomic stability.

We then investigated whether the frequency of chromosome breaks increased at CFSs, by focusing on FRA3B, the CFS that shows the highest frequency of breakage after replication stress in HCT116 cells [43]. To this end, we knocked down Dicer or Drosha by siRNA before inducing replication stress using low doses aphidicolin. Fluorescence *in situ* hybridization (FISH) was performed on metaphase spreads using a FRA3B specific probe. By counting the number of breaks, we found that fragility at FRA3B significantly increases in the absence of both Dicer and Drosha (12,8% in control cells, 26,7% and 19,5% in siDicer and siDrosha cells respectively) (Figure 1E and 1F). To confirm our finding, we also examined FRA3B fragility in Dicer Ex5/Ex5 cells. In line with our previous results, Dicer Ex5/Ex5 cells were more susceptible to breakage at FRA3B after inhibition of replication by aphidicolin, compared to their WT counterparts (Figure 1G; 11% and 22% in WT and Dicer Ex5/Ex5 cells respectively). Taken together these data indicate that Dicer is required for preventing chromosome instability at CFSs.

### Inhibition of Dicer impairs FANCD2 foci assembly without affecting its mono-ubiquitylation

To study the role of Dicer in the response to replication stress, cells were assessed for phosphorylated H2A.X at serine 139 (γH2AX) nuclear foci, an established marker of DNA damage [47]. Surprisingly, immunofluorescence analysis of HCT116 cells after RNAi targeting either Dicer or Drosha showed that inhibition of the Dicer pathway does not affect γH2AX levels after replication stress compared to control cells (Supplementary Figure 2A and 2B). γH2AX staining was also unaffected in aphidicolin-treated Dicer Ex5/Ex5 cells (Supplementary Figure 2C–2E), indicating that the early response to replication stress and DNA damage [48] is not affected by Dicer deficiency.

**Figure 2:**
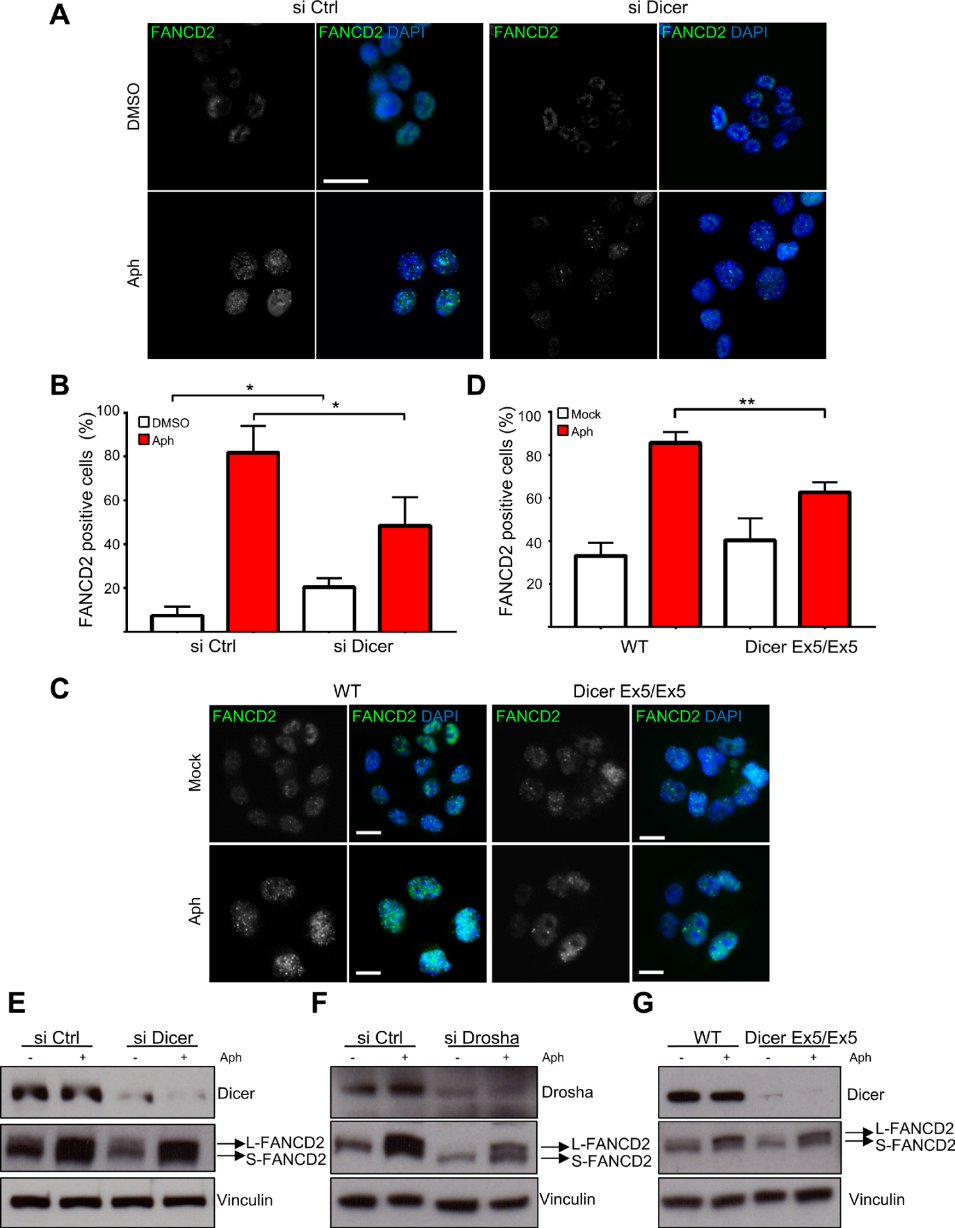
Dicer inhibition prevents FANCD2 foci formation after replication stress without affecting FANCD2 mono-ubiquitylation. (**A**) HCT116 cells were treated with control or Dicer siRNA and then with aphidicolin to induce replicative stress. They were then stained for FANCD2 and analyzed by immunofluorescence microscopy. (**B**) Quantification of the experiments in panel (**A**) showing the percentage of FANCD2 positive cells. Error bars represent the SD of three independent experiments. To score the FANCD2 positive cells a threshold value was calculated on the base of the average number of foci in control cells for each replicate, using Image J. Unpaired *t*-test: ^*^ *p *< 0.05. (**C**) FANCD2 foci formation in WT and Dicer Ex5/Ex5 cells after replication stress induced by aphidicolin. (**D**) Quantification of the experiments in panel (**C**) showing the percentage of FANCD2 positive cells. Error bars represent the SD of three independent experiments. Unpaired *t*-test: ^**^ *p *< 0.005. (**E**) Western blotting showing the levels of FANCD2 mono-ubiquitylation (L-FANCD2) after siRNA-mediated inhibition of Dicer, in the presence or absence of replication stress induced by aphidicolin. Vinculin was used as a loading control. (**F**) Western blotting showing FANCD2 mono-ubiquitylation levels after Drosha inhibition by siRNA. (**G**) FANCD2 mono-ubiquitylation levels in WT and Dicer Ex5/Ex5 cells assayed by western blotting.

We then investigated the foci formation of FANCD2, a protein of the FA pathway that stabilizes replication forks undergoing replication stress [49] and maintain CFS stability throughout S-phase and mitosis [50–52]. As shown in Figure 2A and 2B, the downregulation of Dicer significantly inhibits the formation of FANCD2 foci induced by replication stress. In order to verify these results, we repeated the experiment using the Dicer Ex5/Ex5 cell line and found that FANCD2 foci assembly was also compromised (Figure 2C and 2D).

It has been shown that FANCD2 foci formation is dependent on its mono-ubiquitylation by the FA complex [53]. We therefore set out to investigate whether Dicer pathway is involved in the mono-ubiquitylation of FANCD2. Dicer or Drosha were downregulated by siRNAs and the cells were treated with aphidicolin to induce replication stress. Mono-ubiquitylation of FANCD2, assessed as the ratio between the slow and fast migrating FANCD2 forms by western blotting, remained unaffected by the knock down of both Dicer and Drosha (Figure 2E and 2F). The experiment was repeated using WT and Dicer Ex5/Ex5 cells, confirming the previous result (Figure 2G).

Altogether, the above results indicate that Dicer is not involved in the mono-ubiquitylation of FANCD2, but it promotes FANCD2 foci formation downstream of its mono-ubiquitylation.

### Dicer is essential for activating the S-phase checkpoint after replication stress

**Figure 3:**
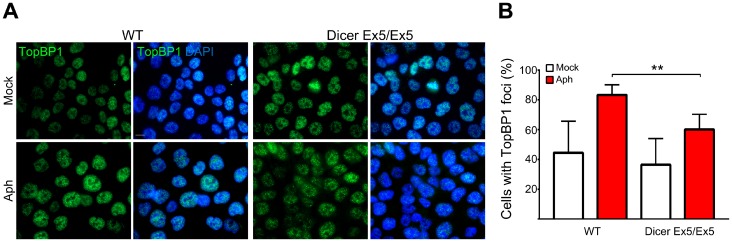
TopBP1 nuclear foci formation induced by replication stress is Dicer-dependent. (**A**) Immunofluorescence and microscopy analysis of mock (DMSO) or aphidicolin-treated WT and Dicer Ex5/Ex5 HCT116 cells stained for TopBP1. (**B**) Quantification of the experiments in panel (**A**) showing the percentage of TopBP1 positive cells. Error bars represent the SD of three independent experiments. To score the TopBP1 positive cells a threshold was applied on the base of signal intensity in control cells for each replicate. Unpaired *t*-test: ^**^ *p *< 0.005.

**Figure 4:**
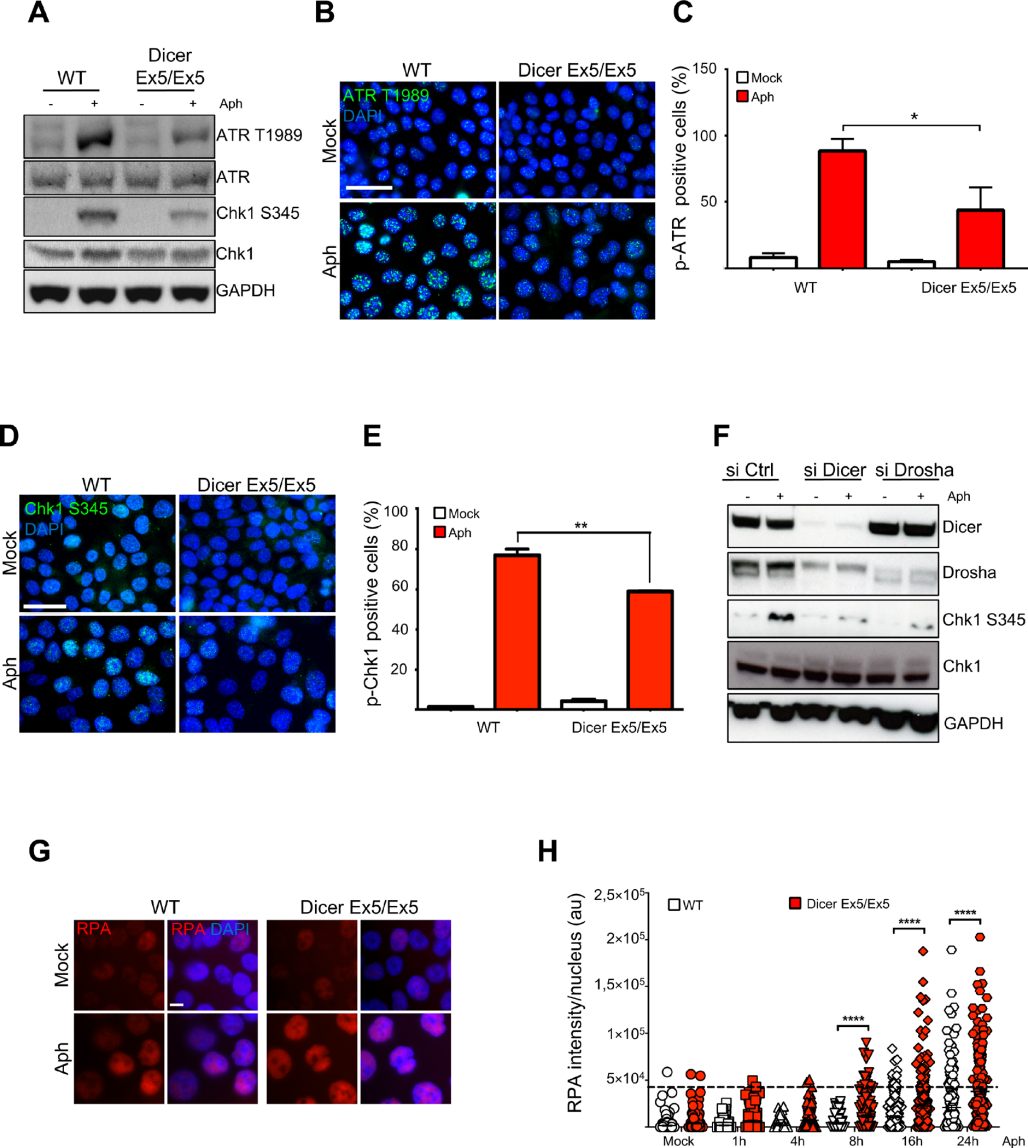
Inhibition of Dicer impairs the S-phase checkpoint. (**A**) WT and Dicer-deficient HCT116 cells were treated with DMSO (−) or aphidicolin (+) and then assayed for several markers of the S-phase checkpoint by Western blotting. GAPDH was used as loading control. Total ATR and Chk1 levels were assayed and used as controls for the changes in phospho-ATR and phospho-Chk1 levels respectively. (**B**) Phosphorylation of ATR was assayed by immunofluorescence in WT and Dicer Ex5/Ex5 cells treated with DMSO (Mock) or aphidicolin (left panel). DNA was stained with DAPI. The cells were analyzed by microscopy. (**C**) Quantification of the experiments in panel (**B**), showing the percentage of phospho-ATR positive cells. Error bars represent the SD of three independent experiments. A threshold was applied on the base of the signal intensity in control cells for each replicate. Unpaired *t*-test: ^*^ *p *< 0.05. (**D**) Immunofluorescence experiment showing inhibition of aphidicolin-induced Chk1 phosphorylation at serine 345 after aphidicolin treatment in the absence of functional Dicer. (**E**) Quantification of the experiments in panel (**D**), showing the percentage of phospho-Chk1-positive cells. Error bars represent the SD of three independent experiments. A threshold was applied on the base of the signal intensity in control cells for each replicate. Unpaired *t*-test: ^**^ *p *< 0.005. (**F**) Western blotting showing inhibition of Chk1 phosphorylation in the absence of Dicer or Drosha. HCT116 cells were treated with siRNA against Dicer or Drosha and then treated with DMSO (−) or aphidicolin (+) to activate the replication checkpoint. (**G**) Representative immunofluorescence experiment showing the increase of nuclear RPA after aphidicolin treatment in the absence of functional Dicer. (**H**) Quantification of nuclear RPA signal intensity at the indicated time points after aphidicolin addition. Error bars represent the SEM of three independent experiments. Mann-Whitney test: ^****^ *p *< 0.0001.

To further investigate the mechanisms underlying Dicer function in genomic stability after replication stress, we analyzed the behavior of TopBP1, another protein fundamental for replication stress response and CFS maintenance [54, 79]. Similar to FANCD2, TopBP1 foci formation was significantly reduced in the Dicer-deficient cells (Figure 3A and 3B). Since TopBP1 recruitment is essential for activating the ATR-dependent checkpoint [16], we then compared the efficiency of the S-phase checkpoint signaling in WT and Dicer Ex5/Ex5 cells treated with aphidicolin. Checkpoint activation was analyzed by quantifying the levels of phosphorylated Chk1 (at serine 345) and ATR (at threonine 1989), established marks of the S-phase checkpoint [55–57]. Analysis by western blotting showed that the levels of both phosphorylated Chk1 and ATR after aphidicolin treatment were reduced in absence of a fully functional Dicer pathway, while total levels of Chk1 or ATR were not affected (Figure 4A), indicating that the S-phase checkpoint activation was significantly inhibited.

We then set out to confirm the role of Dicer in regulating the S-phase checkpoint by performing immunofluorescence analysis. In accordance to the previous western blotting results, we observed a decrease in the focal staining of phosphorylated ATR in absence of functional Dicer, in response to replication stress (Figure 4B and 4C). Moreover, immunofluorescence analyses showed a strong inhibition of Chk1 phosphorylation after aphidicolin treatment in the absence of functional Dicer (Figure 4D and 4E).

Likewise, the levels of Chk1 phosphorylation on serine 345 in response to replication stress determined by western blotting (Figure 4F) were reduced after RNAi-mediated inhibition of the Dicer pathway. The decreased Chk1 phosphorylation after replication stress was also observed in Dicer-depleted HeLa cells (Supplementary Figure 3A).

To further support the role of Dicer in replication stress response and its impact on DNA damage, we analyzed the levels of RPA2 phosphorylation on serine 33 (a specific target of ATR). The aphidicolin treatment induced a sharp increase of RPA2 phosphorylation at 16h in WT cells, indicating the activation of ATR checkpoint. On the contrary, RPA2 phosphorylation was not induced at the same level in Dicer Ex5/Ex5 cells, confirming that the ATR-dependent checkpoint was not fully activated (Supplementary Figure 3B). Defective checkpoint activation following replication stress leads to the accumulation of single-stranded DNA [11]. Therefore, we compared the levels of nuclear RPA in WT and Dicer deficient cells at different time points following aphidicolin treatment. Immunofluorescence analysis showed higher amounts of nuclear RPA in Dicer Ex5/Ex5 cells starting from 8h after treatment. (Figure 4G and 4H). These data strongly suggest that the ATR-Chk1 dependent checkpoint signaling is dysfunctional in the absence of functional Dicer or Drosha.

### Inhibition of Dicer prevents the cell cycle arrest induced by replication stress

**Figure 5:**
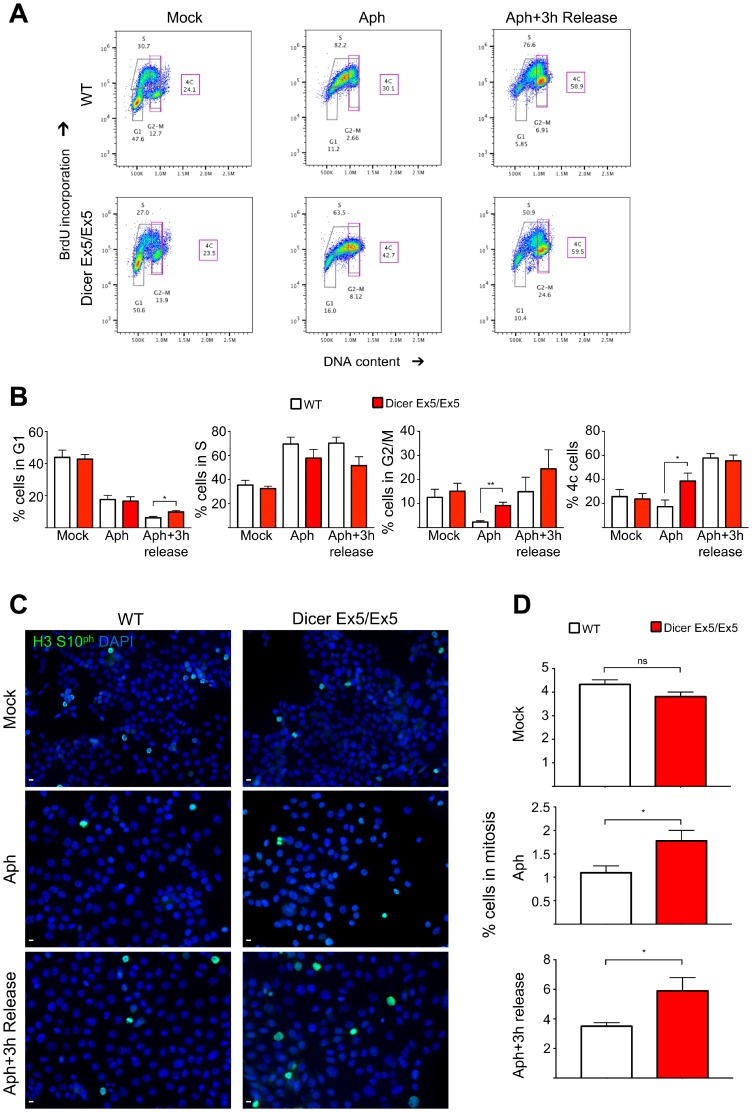
Inhibition of Dicer impairs aphidicolin-induced cell cycle arrest in S phase. (**A**) Representative cell cycle profiles of WT and Dicer-deficient HCT116 cells stained with BrdU after 24h aphidicolin treatment and 3 h after removal of aphidicolin (3 h release). Cells were incubated with anti-BrdU antibodies, stained with propidium iodide (PI) and analyzed by flow cytometry. The proportion of cells in the different phases of the cell cycle is indicated. (**B**) Quantification of the cells in each cell cycle phase and of cells with 4C DNA content (late-S and G2-M cells). Error bars represent the SEM of four independent experiments. Unpaired *t*-test: ^*^ *p *< 0.05, ^**^ *p *< 0.005. (**C**) Dicer-deficient cells escape the S-phase arrest and enter mitosis after aphidicolin treatment. WT and Dicer-deficient HCT116 cells were treated with aphidicolin, which was then removed (3 h release). Cells were analyzed by immunofluorescence and microscopy, after staining for phosphorylated Histone H3 (S10), a known marker of mitosis. (**D**) Quantification of the experiments in panel (**C**). Error bars represent the SEM of four independent experiments. Unpaired *t*-test: ns not significant, ^*^ *p *< 0.05.

To confirm the impairment of the S-phase checkpoint after inhibition of Dicer, WT and Dicer Ex5/Ex5 HCT116 cells were treated for 24 h with aphidicolin to induce replication stress before being released for 3h in normal medium. Cell cycle profiles were then analyzed by flow cytometry. BrdU and PI staining showed that WT cells treated with aphidicolin arrested in S phase with only 2.3% of cells entering G2-M, likely as a consequence of fork slowing and checkpoint activation to give time to repair any damage induced by replication stress. By contrast, a significantly higher percentage of Dicer-deficient cells (9.1%) proceeded to G2-M (Figure 5A and 5B). We also quantified the fraction of cells with duplicated genome content (late S and G2-M cells, indicated as 4C DNA content in Figure 5A and 5B). Aphidicolin treatment led to a strong inhibition of genome duplication in WT HCT116 cells, with only 17.4% of cells having 4C DNA (Figure 5B). On the other hand, in Dicer Ex5/Ex5 cells, aphidicolin treatment did not lead to a firm cell cycle arrest, with the vast majority of cells progressing through S-phase and G2-M (38.6% of the cells exhibit a 4C DNA content) (Figure 5B). When WT HCT116 cells were released from aphidicolin for 3 hours, they re-entered the cell cycle proceeding to late S and starting to enter G2-M phase (Figure 5A and 5B). Dicer Ex5/Ex5 cells instead proceeded from late S to G2-M and from G2-M to the next G1 phase (Figure 5A and 5B). Accordingly, the percentage of cells in both late S and G2-M phase (cells with 4C DNA content) was comparable between WT HCT116 and Dicer Ex5/Ex5 cells (Figure 5B). However, the percentage of G2-M cells tended to be higher in Dicer Ex5/Ex5 cells than in WT HCT116 (24.4% vs 14.9%) (Figure 5B).

To examine whether these cells reach mitosis after replication stress, we stained the cells for Histone H3 phosphorylated at serine 10, a well-known marker of mitotic chromatin condensation (Figure 5C and 5D). As expected, immunofluorescence analysis showed that aphidicolin treatment leads to a strong inhibition of mitotic entry in WT cells, as consequence of their functional S-phase checkpoint that keeps them from progressing through the cell cycle. Conversely, as quantified in Figure 5D, a significant fraction of Dicer Ex5/Ex5 cells escaped the cell cycle arrest and entered into mitosis. Moreover, this analysis showed that 3 hours after release from aphidicolin, a higher proportion of Dicer Ex5/Ex5 cells are in mitosis when compared to WT cells (5.1% vs 2.9%). Overall, these data show that Dicer is required for arresting the cell cycle in S phase after replication stress, and to prevent cells from entering mitosis with unrepaired DNA damage or under-replicated DNA.

### Dicer deficiency increases mitotic transmission of DNA damage in response to replication stress

**Figure 6:**
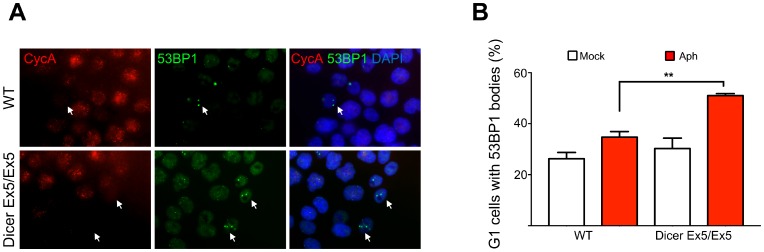
Dicer inhibition causes increase in 53BP1 nuclear bodies. (**A**) Immunofluorescence and microscopy analysis of mock or aphidicolin-treated WT and Dicer Ex5/Ex5 cells stained for 53BP1 and Cyclin A. (**B**) Quantification of the experiment in panel (**A**), showing the percentage of Cyclin A negative cells with 53BP1 bodies. Error bars represent the SEM of three independent experiments. Unpaired *t*-test: ^**^ *p *< 0.005.

To further confirm the involvement of Dicer in preserving genome stability during replication stress, we tested the formation of 53BP1 bodies, nuclear structures that assemble around DNA lesions generated from the under-replicated DNA persisting in the previous mitosis [58]. WT and Dicer Ex5/Ex5 cells were treated with aphidicolin and then analyzed by immunofluorescence using antibodies directed against 53BP1 and Cyclin A, to quantify the G1 cells with 53BP1 bodies. As shown in Figure 6A and 6B, the absence of functional Dicer led to a significant increase in the number of G1 (Cyclin A negative) cells with 53BP1 bodies (50,9%) as compared to WT cells (34,7%), confirming that Dicer function is required to prevent the formation of replication induced DSB and to limit their transmission to daughter cells.

## DISCUSSION

In this study, we examined the role for Dicer in the DNA damage response induced by replication stress. To this end, we focused our work on CFSs, which are particularly prone to breakage following replication stress. Our data clearly show that inhibition of the Dicer pathway, either by siRNA targeting Dicer and Drosha or using a Dicer-deficient cellular model, prevents the efficient and timely resolution of stalled replication forks, leading to enhanced chromosome fragility and accumulation of 53BP1 bodies. This effect can be attributed to the involvement of Dicer in the activation of the replication checkpoint since the S-phase checkpoint was weakened in the absence of a functional Dicer protein. The deficient checkpoint signaling led to a prominent inhibition of cell cycle delay in S-phase and uncontrolled progressing of cells with under-replicated DNA in G2-M phase. Indeed, this S-phase arrest is essential to give cells time to resolve the stalled replication forks, avoiding their collapse and subsequent chromosome breakage during mitosis [59–62]. However, whether this role of Dicer is mediated via its miRNA synthesis function or via a non-canonical miRNA-independent function [63, 64] remains to be determined.

FANCD2 is a protein of the FA pathway that has an important role in preventing genomic instability in conditions of replication stress. Our study shows that the Dicer-pathway promotes FANCD2 assembly into nuclear foci following replication stress. However, we show that FANCD2 is efficiently mono-ubiquitylated in the absence of Dicer, suggesting that miRNA pathway acts on FANCD2 response to replication stress (foci formation) through another mechanism. Noteworthy, deficiency of USP1 (the FANCD2 deubiquitylating enzyme) leads to a constitutively mono-ubiquitylated FANCD2, yet impairs its foci formation, showing that FANCD2 mono-ubiquitylation is necessary but not sufficient to induce FANCD2 foci formation [65]. In addition, it has been shown that the role of FANCD2 in controlling replisome function is independent from its mono-ubiquitylation by the FA core [49]. One possibility is that Dicer or small RNAs promote FANCD2 mobilization or retention to the site of replication fork stalling. Future work will be needed to further clarify this important issue. Apart from FANCD2, cells lacking functional Dicer treated with aphidicolin also fail to induce the accumulation of TopBP1 foci, an essential ATR activator. The interaction between TopBP1 and the ATR-ATRIP complex at stalled replication forks is essential for activating the S-phase checkpoint [16]. On the other hand, it has been shown that the mono-ubiquitylation of FANCD2 is TopBP1-independent, which is consistent with the fact that mono-ubiquitylation of FANCD2 remains unaffected in the absence of Dicer [66].

**Figure 7:**
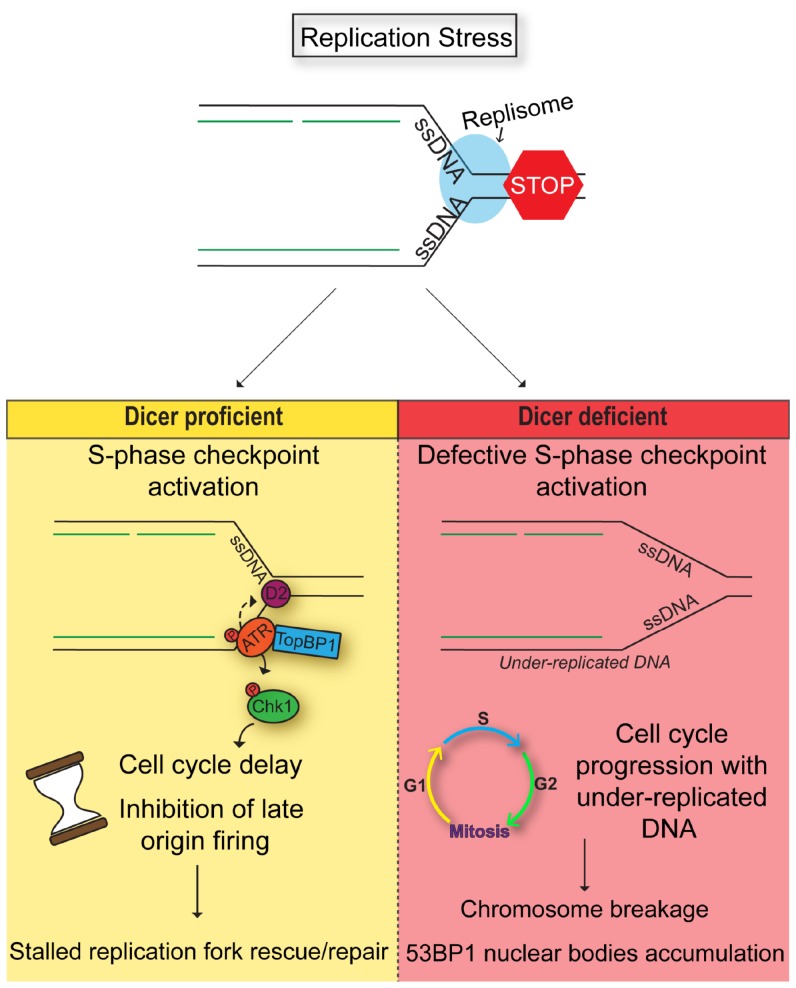
Model. Dicer inhibition causes a defective S-phase checkpoint activation in response to DNA replication stress, allowing cells to enter mitosis despite the presence of incompletely replicated DNA. As a result, cells undergo chromosomal breakage and eventually formation of 53BP1 nuclear bodies in the next G1.

Our study demonstrates a role for Dicer in the DNA damage response induced by replication stress in human cells. Treatment of cells with mild doses of aphidicolin leads to replication stress that, in the presence of functional Dicer, activates the S-phase checkpoint. This checkpoint has an important function in preventing the unscheduled activation of late replication origins, that often co-localize at CFSs [1, 2, 10, 67, 68]. Moreover, the S-phase checkpoint results in cell cycle arrest, giving the cells time to resolve stalled forks and prevent further induction of DNA damage. On the other hand, in the absence of Dicer, the S-phase checkpoint is not fully functional. This could allow unrestrained origin activation and DNA synthesis at late replicating regions, including CFSs, and at the same time preventing cell cycle arrest. Consequently, cells proceed into mitosis with under-replicated DNA resulting in chromosome breakage and DNA damage accumulation in daughter cells (Figure 7).

Our data show that Dicer has a function in the activation of the ATR pathway, as Dicer deficient cells fail to phosphorylate ATR and its main effectors RPA2 and Chk1, without affecting H2A.X phosphorylation. This suggests that Dicer acts upstream of the ATR-Chk1 pathway preventing unscheduled origin firing and limiting ssDNA accumulation (Figure 4), which can lead to replication catastrophe upon stringent replication stress [69]. Noticeably, this may explain why a partial suppression of Dicer function promotes tumor development yet its complete loss is not tolerated in cancer cells that have to sustain high levels of replication stress [70]. The specific mechanism by which Dicer functions to control the S-phase checkpoint and prevent chromosome breakage remains unknown. Nevertheless, it has been shown that, in the case of double-strand breaks, small Dicer-dependent RNAs act by recruiting chromatin modifiers, which add chromatin marks that are used as guides to attract repair factors at the sites of breaks [29, 31]. In fission yeast, Dicer was shown to act by unloading RNA Pol II from chromatin, to avoid transcription-replication collisions [40] that otherwise would lead to chromosomal damage [71]. Another study has that in response to DNA damage induced by UVs, miRNAs can target CDC25A, one of the main effectors of the ATR-Chk1 pathway involved in the intra-S phase arrest [72]. It is also widely accepted that replication stalling can lead to the functional uncoupling of helicase and polymerase complexes. In this context, the unwinding of the double stranded DNA produces long stretches of single-stranded DNA (ssDNA) [73]. These stretches of ssDNA could represent docking sites for RNA-mediated epigenetic modifications that are necessary in the response to replication stalling. Dicer products transcribed from genomic regions mapping to CFSs could bind specifically to those chromosomal regions by base complementarity and function as guides for chromatin modifiers. miRNAs could also bind to single-stranded DNA to prevent the formation of complex secondary structures that are prone to breakage. Alternatively, Dicer may also have an RNA-independent function in regulating the S-phase checkpoint by controlling the binding of DNA repair proteins at stalled replication forks. Since CFS are particularly prone to undergo replication-transcription collisions and R-loop formation [4], Dicer and Drosha may be involved in dynamic processing of these structures [41], allowing on one side a proper checkpoint activation and, on the other, stable chromatin association of proteins, like FANCD2, that stabilize the forks and promote R-loop resolution [49, 51, 74–76]. These possibilities remain to be investigated, in order to identify the exact mechanism by which Dicer maintains genome stability upon replication stress.

## MATERIALS AND METHODS

### Cell lines and treatments

All experiments were performed using the colorectal cancer cell line HCT116, except for the experiments in Supplementary Figure 3A performed in HeLa cells (ATCC). Dicer Ex5/Ex5 cells and their WT counterparts were kind gifts from Dr Bert Vogelstein [45]. Cells were grown in McCoy’s 5A medium (Life Technologies), supplemented with 10% FBS (Eurobio) and antibiotics (Life Technologies). Replication stress was induced by treating cells with aphidicolin at a concentration of 0.7 μM for 20-24 h. Mock cells were treated with the same volume of DMSO.

### siRNA treatments

Cells were transfected with siRNA pools targeting Dicer (Dharmacon, #M-003483-00-0005) or Drosha (Dharmacon, #M-016996-02-0005) using Interferin (Polyplus), following the manufacturer’s instructions. For mock treated cells, a non-targeting pool of siRNAs was used (Dharmacon, #D-001206-13-05). Cells were treated with siRNA pools for 60 h (Dicer) and 84 h (Drosha), and were then either collected for analysis or treated with aphidicolin (as described above), to induce replication stress.

### Western blotting

Cells were collected, washed with PBS, and resuspended in 2.5 volumes of lysis buffer (50 mM Tris.HCl pH 7.5, 20 mM NaCl, 1 mM MgCl_2_, 0.1% SDS) supplemented with a cocktail of protease and phosphatase inhibitors (Life Technologies or Roche) and benzonase (0.025 units/μl, Novagen). Samples were incubated on a shaking platform at room temperature for 15 min and then centrifuged at 16,000 g for 20 min at 4° C. The protein supernatants were collected and protein concentrations were measured using the BCA protein assay kit (Pierce) or the Bradford assay (Biorad). 10–40 μg of total protein extracts from each sample were resolved on 7%, 3–8% and 4–12% gradient sodium dodecyl sulfate-polyacrylamide gels (Life Technologies or Biorad). Protein samples were then transferred onto nitrocellulose or PVDF membranes (Life Technologies or Biorad), which were then blocked for 3–5 h in blocking solution (5% milk powder –0.1% Tween in PBS) at room temperature. The membranes were incubated with primary antibodies overnight at 4 °C, washed three times with PBS-0.1% Tween, incubated with secondary antibodies for 1 h at room temperature, and finally washed three times with PBS-0.1% Tween. Blots were visualized using either an ECL assay (Ozyme or Advansta) or an immunofluorescence detection protocol, following the manufacturer’s instructions.

The primary antibodies used were the following: anti-Dicer (Abcam, #ab14601), anti-Drosha (Abcam, #ab12286), anti-Piwi (Abcam, #ab12337), anti-GADPH (Abcam, #ab8245), anti-chk1 (Cell signaling, #2360), anti-phospho-chk1 (serine 345) (Cell Signaling, #2348), anti-ATR (Cell signaling, #2790), anti-phospho-ATR (threonine 1989) (Genetex, #GTX128145), anti-FANCD2 (Santa Cruz, #sc-20012), anti-FANCD2 (Novus, #NB-100-182), anti-TopBP1 (Abcam, #ab2402), anti-Vinculin (Abcam, #ab180581), anti-Lamin A/C (Santa Cruz, #sc-7292), anti-RPA2 (Calbiochem, #NA18), anti-phospho-RPA2 (serine33) (Bethyl, #A300-246A, a kind gift of Dr. Stéphane Koundrioukoff) and anti-GFP (Roche, #11814.460001). Horseradish peroxidase-conjugated antibodies (Jackson Immunoresearch, #85100 and #81283; Bethyl, #A90-1375 and #A120-1088) were used as secondary antibodies for ECL detections. Alexa Fluor 750- conjugated Goat anti-mouse (#A21037) and Goat-anti-Rabbit (#A21039) (Thermofisher Scientific) were used as fluorescent secondary antibodies and the corresponding acquisitions were then performed using GBOX (Ozyme).

### Metaphase spreads

After aphidicolin treatment, cells were washed with medium and left in the incubator in the presence of 10 μM nocodazole for 6 h. Cells were then trypsinized, transferred into tubes and centrifuged. Each cell pellet was then resuspended in 10 ml of warm (37° C) hypotonic buffer (10 mM KCl, 15% FBS) for 10 min at 37° C. Cold (4° C) fixation buffer (25% acetic acid, 75% ethanol) was then added (0.5 ml per sample) at 37° C and cells were then immediately centrifuged. Cell pellets were washed 5 times with 6 ml of cold fixation buffer and were finally kept in a small volume (100–200 μl) of cold fixation buffer. The samples were then used to prepare metaphase spreads by transferring a single drop of fixed cells with a Pasteur pipette onto a wet microscope slide. For each experiment, at least 80 metaphases per condition were analyzed by visual inspection of microscopy images using the Image J, in a non-blinded manner.

### Probe preparation

200 μl of BAC DNA (clone RP11-147N7, located in FRA3B) were sonicated using the Bioruptor UCD-300 (20–25 one-min cycles), at a concentration of 25 ng/μl. The sonicated DNA was then subject to labelling by using the Platinum Bright kit by Kreatech, following the manufacturer’s instructions.

50 ng of labelled probe were mixed with 50 μg of herring sperm DNA (Life Technologies) and 10 μg of COT human DNA (Kreatech), in a total volume of 50 μl. DNA was then precipitated by adding 10 % sodium acetate and 2.5 volumes of pure ice-cold ethanol and incubating the sample at –80° C for 1–2 h. The samples were then centrifuged (13,000 rpm, 15 min, 4° C) and pellets were washed with 70% ethanol (300 μl). Samples were centrifuged again and pellets were then left to dry. They were suspended in 10 μl of hybridization solution (40 % formamide, 10% dextran sulfate, 2× SSC), denatured at 95°C for 5 min and kept on ice.

### Fluorescence *in situ* hybridization (FISH)

The hybridization reaction has been previously described [77]. In brief, the slides with metaphase spreads were incubated at 80° C for 1 min, in the presence of denaturing solution (70% formamide, 2x SSC). The slides were then successively washed in 70%, 90% and 100% ethanol for 3 min and were left to dry. The denatured probe was then added on top of the slides, with the help of a coverslip, and the slides were incubated in a humid chamber at 37° C overnight. The slides were then washed in washing buffer (0.5× SSC, 0.1% SDS) for 5 min at 65° C, then with PBS for 5 min at room temperature and were finally analyzed by fluorescence microscopy, after adding a drop of Vectashield mounting medium (Vector laboratories, # H-1200) per slide.

### Immunofluorescence and microscopy

Cells were grown on coverslips and then washed twice with PBS before fixation with formaldehyde (4%) for 10-15 min at room temperature. For phospho-Histone3 serine 10 antibody (Cell Signaling, #9701), the following protocol was performed. Cells were washed with PBS and incubated in blocking buffer (0.5% Igepal, 5% milk powder, 1% FBS) for 30 min at room temperature. After being washed with PBS, cells were incubated overnight at 4° C with primary antibodies, in a buffer containing 5% milk powder and 1% FBS. Coverslips were washed with PBS and then incubated in the same buffer, containing secondary antibodies (goat anti-rabbit FITC, #4050-02 from Southern Biolabs and Alexa Fluor 568 goat anti-rabbit, #A11011 from Life Technologies), for 45-60 min at room temperature. Cells were washed twice with PBS, once with distilled water and were then transferred on microscope slides containing 20 μl of Vectashield mounting medium.

For phospho-Chk1 serine 345 (Cell Signaling, #2348), TOPBP1 (Santa Cruz, sc-32923), 53BP1 (Abcam, #ab36823), FANCD2 (Abcam, #ab2187), phospho-H2AX (serine 139) (Cell Signaling, #2577), Cyclin A2 (Abcam, #ab38) and RPA2 (Calbiochem, #NA18) antibodies, cells were fixed as above and then permeabilized using PBS-0.5% Triton X-100 for 15 min, before being blocked with blocking solution (PBS-3% BSA-0.05% Tween) for 1-3 h at room temperature. Coverslips were then incubated with the primary antibodies (diluted in blocking solution) overnight at 4° C. Coverslips were then washed 3 times with PBS and incubated with the corresponding secondary antibodies (goat anti-rabbit FITC, #4050-02 from Southern Biolabs; Alexa Fluor 568 goat anti-rabbit #A11011, Alexa Fluor 488 goat anti-rabbit #A21206 and Alexa Fluor 594 goat anti-rabbit #A21207 from Life Technologies) diluted in blocking solution, for 45–60 minutes at room temperature. After being washed three times (10 min each) in PBS and once with distilled water, cells were mounted on microscope slides containing Vectashield mounting medium or 10 μl of DAPI fluoromount medium from Southern Biotech (#0100-20).

For phospho-ATR (threonine 1989) antibody, cells were fixed with 100% methanol for 5 min at −20° C, permeabilized using 0.1% Tween-20 and washed using PBS-0.1% tween-20. All slides were analyzed using a Zeiss epifluorescence microscope.

For each experiment, at least 100 cells per condition were scored by visual inspection of microscopy images using the Image J, in a non-blinded manner.

### Flow cytometry

Cells were treated with 10 μM of BrdU (Sigma) for 30 min before harvesting. Cells were trypsinized, washed with PBS, and centrifuged 10 min at 800 rpm. Cells were then fixed in ice-cold 70% ethanol and incubated at 4°C overnight, as described [43, 78]. Cell were centrifuged 10 min at 1000 rpm and the pellet resuspended in denaturing solution (0.1M HCl) on ice for 10 min. Cells were then diluted in cold distilled water and centrifuged 10 min at 1000 rpm. Cell pellets were resuspended in water, boiled for 10 min and incubated on ice for 15 min. Cells were permeabilized with 0.5% Triton X-100 in PBS, centrifuged and incubated with BrdU antibodies (DAKO, #M0744) in blocking solution (0.1% BSA in PBS) for 1 h at room temperature. Cells were washed with cold PBS and incubated with secondary antibodies (goat anti-mouse FITC-conjugated antibodies from Thermo Fisher Scientific, #626511) for 45 min at room temperature in the dark. Finally, cells were resuspended in PI/RNase staining buffer (BD Bioscience, # 550825) and analyzed by flow cytometry (BD Accuri C6). The discrimination of cell doublets from singlets was made by plotting the DNA dye (PI) channel Width versus Area in a dot plot graph. The gating was done by separating BrdU positive cells (active S-phase) from the negative ones, and by distinguishing 2n and 4n content cells (G1 and G2/M phase respectively) on the base of DNA content (PI staining).

### Statistical analysis

Statistical analysis of the FISH data was performed using ANOVA and Student’s *t*-test (two-tailed). For all other experiments, statistical significance was calculated by using the test indicated in the legend. Statistical significance is indicated with ns (not significant), ^*^(*p* < 0.05), ^**^(*p* < 0.005), ^***^(*p* < 0.001) or ^****^(*p* < 0.0001).

## SUPPLEMENTARY MATERIALS


