# Supplementary material for: Dicer prevents genome instability in response to replication stress
Source: Oncotarget. 2019 Jul 9;10(43):4407–23. doi: 10.18632/oncotarget.27034 (PMC6633883; doi:10.18632/oncotarget.27034)
Supplement: Supplementary file 1 [file oncotarget-10-4407-s001.pdf]

Dicer prevents genome instability in response to replication stress

SUPPLEMENTARY MATERIALS

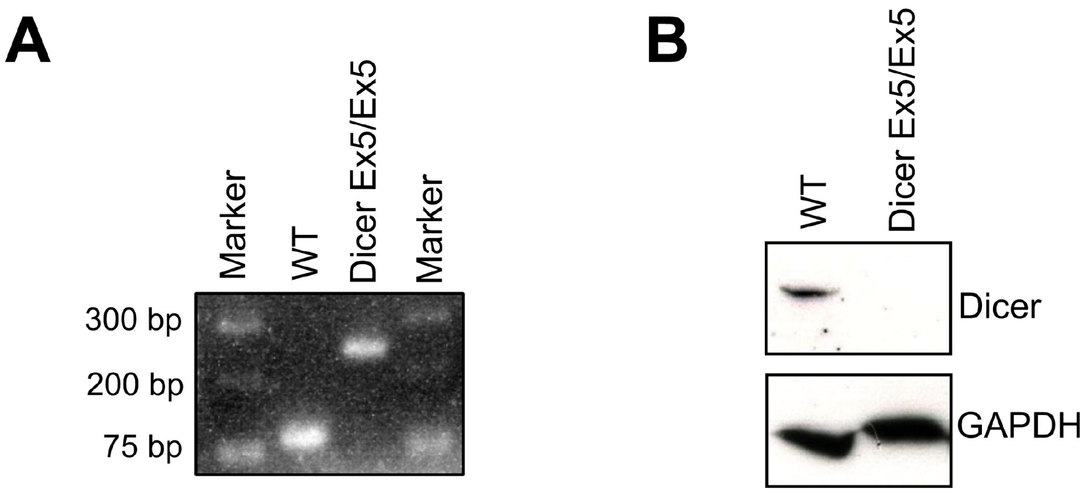

Supplementary Figure 1: Genotype verification of the Dicer Ex5/Ex5 HCT116 cells. (**A**) PCR-based assay to verify the 129-bp insertion in exon 5 of Dicer gene in Dicer Ex5/Ex5 HCT116 cells. (**B**) Western blot showing absence of intact Dicer protein in Dicer Ex5/Ex5 cells.

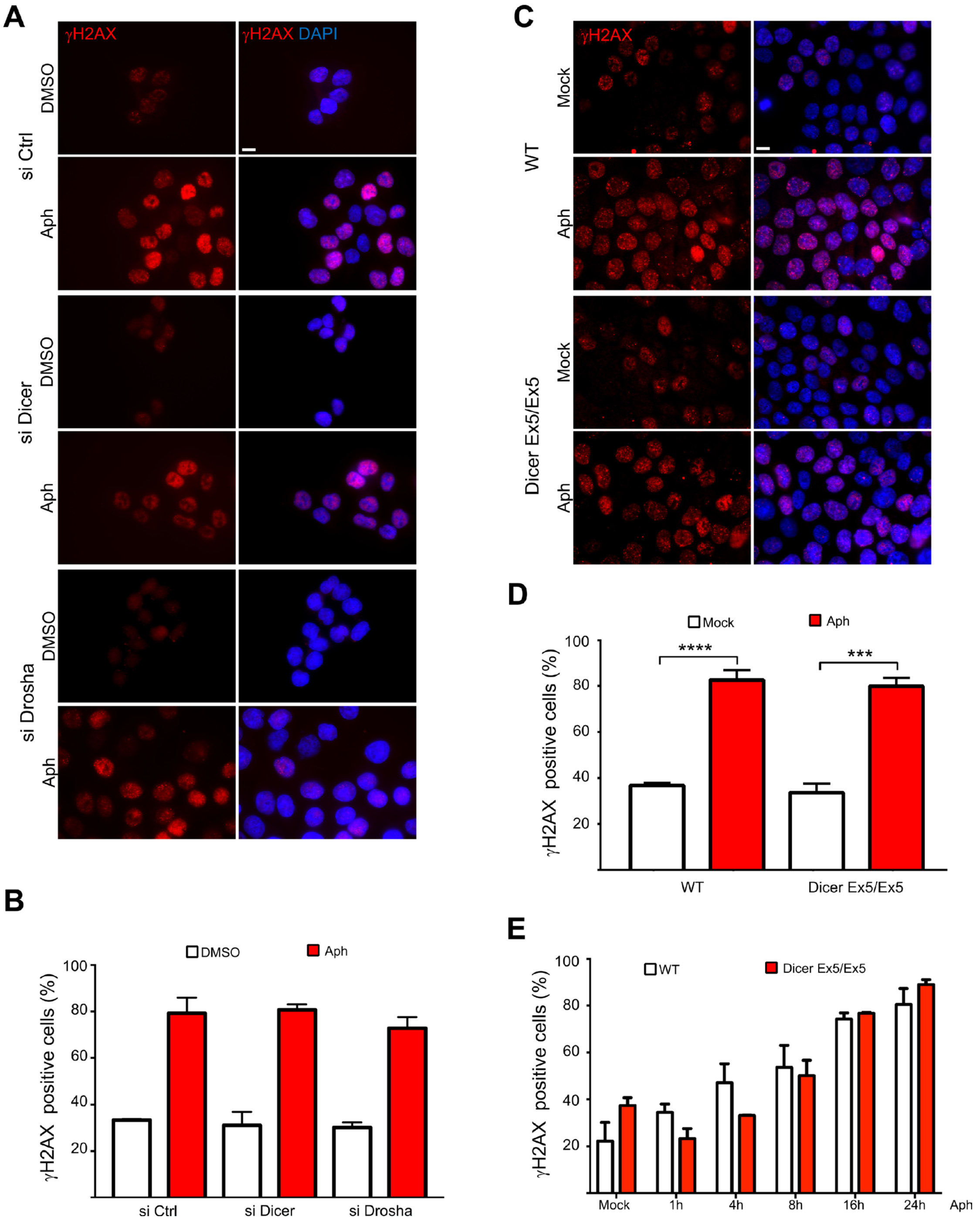

Supplementary Figure 2: Inhibition of Dicer does not affect γH2AX staining induced by replication stress. (**A**) Immunofluorescence staining showing γH2AX positive nuclei in si Ctrl, si Dicer and si Drosha cells in the absence or presence of aphidicolin-induced replication stress. (**B**) Quantification of the experiments in panel (**A**), showing the percentage of γH2AX positive cells. Error bars represent the SD of two independent experiments. (**C**) HCT116 WT and Dicer Ex5/Ex5 treated or not with aphidicolin and analyzed for γH2AX staining by immunofluorescence. (**D**) Quantification of the experiments in panel (**C**), showing the percentage of γH2AX positive cells. Error bars represent the SD of three independent experiments. Unpaired *t*-test: $^{***}p < 0.001$, $^{****}p < 0.0001$. (**E**) Histogram showing the quantification of γH2AX positive nuclei in HCT116 WT and Dicer Ex5/Ex5 cells at different time points after aphidicolin treatment. Error bars represent the SEM of three independent experiments. Mann-Whitney test: not significant.

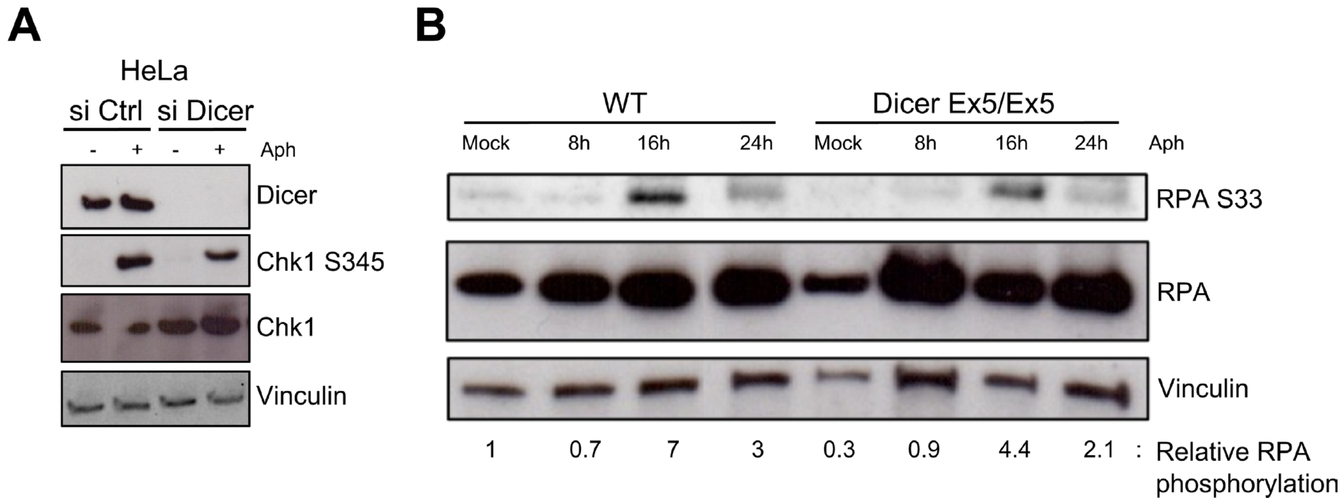

Supplementary Figure 3: (**A**) Western blotting showing inhibition of Chk1 phosphorylation in the absence of Dicer. HeLa cells were transfected with control siRNA or siRNA against Dicer and then were left untreated (−) or treated with aphidicolin (+) for 24 h. (**B**) Western blot quantification of RPA S33 phosphorylation relative to total RPA in HCT116 WT and Dicer Ex5/Ex5 cells mock-treated or treated with aphidicolin for the indicated time points. Values are normalized with respect to the mock-treated WT sample.
